# Pyoderma Gangrenosum as a Presenting Feature of Undifferentiated Spondyloarthropathy with Erosive Inflammatory Arthritis

**DOI:** 10.1155/2020/1848562

**Published:** 2020-03-26

**Authors:** Jaspreet Kaler, Sandra Sheffield, Myint Thway, Karishma Ramsubeik, Gurjit Kaeley

**Affiliations:** ^1^Department of Rheumatology, University of Florida Health, 653 W 8^th^ Street, Jacksonville, 32209 Florida, USA; ^2^Department of Medicine, University of Florida Health, 653 W 8^th^ Street, Jacksonville, 32209 Florida, USA

## Abstract

Pyoderma gangrenosum is a rare inflammatory condition with varying clinical presentations and severity. It is commonly seen in association with an underlying condition, most common of which is inflammatory bowel disease. We report a case of a 26-year-old male who came to the emergency department with increasing lower extremity ulcers, intermittent hematochezia, and pain in the small joints of his hands. After excluding a broad list of differentials for lower extremity ulcers, the diagnosis of pyoderma gangrenosum was made. He was also found to have erosive changes at multiple proximal interphalangeal joints and jug-like syndesmophytes at T12 and L1 on CT scan. Although there was evidence of a spondyloarthropathy, there was no evidence of inflammatory bowel disease on colonoscopy, psoriasis, or sexually transmitted infections. After multiple failed trials of medications including azathioprine and sulfasalazine, 4 weeks of Adalimumab resulted in rapid healing of pyoderma gangrenosum lesions and improvement in his synovitis. Coupled together, this suggests a diagnosis of pyoderma gangrenosum associated with undifferentiated spondyloarthropathy and erosive inflammatory arthritis. This case is suggestive of spondyloarthropathy going underdiagnosed and untreated in other patients with pyoderma gangrenosum as lower extremity ulcerations can be the primary complaint for seeking treatment. Although rare, axial spondyloarthropathy associated with pyoderma gangrenosum should be kept as an associated differential diagnosis when faced with pyoderma gangrenosum.

## 1. Introduction

Pyoderma gangrenosum is a rare inflammatory condition with varying clinical presentations and severity. Typically, it presents as a papule or pustule that rapidly evolves to an ulcerative lesion with irregular, erythematous to violaceous edges. Pathergy is a characteristic feature of pyoderma gangrenosum as lesions often occur at sites of trauma. It has an estimated annual incidence of 3–10 cases per million people [1]. It can affect people of any age, but incidence peaks between the ages of 20 and 50 years old and affects men and females equally [2]. Although pyoderma gangrenosum can be found independently, it is more commonly found in association with an underlying disease. In fact, up to 75% of pyoderma gangrenosum cases are associated with inflammatory bowel diseases, inflammatory arthritis, or hematological disorders [2]. Of the accompanying disorders, inflammatory bowel diseases, such as Crohn's disease and ulcerative colitis, are the associated disease in 65% of cases [2]. We present a case of pyoderma gangrenosum as a presenting feature of undifferentiated spondyloarthropathy, with erosive inflammatory arthritis, without any evidence of inflammatory bowel disease.

## 2. Case Report

A 26-year-old Hispanic male with no significant past medical history came to the emergency department complaining of multiple wounds that gradually developed over the past 3 years. He reported a wound at the vertex of his scalp, posterior aspect of his neck, and on bilateral lower extremities. He reported the wounds initially started as small pimples, with progression in size and pain severity. The patient also reported serosanguineous drainage from his wounds, hematochezia, and pain in the small joints of his hands bilaterally, 9/10 in severity, associated with swelling and stiffness. The patient reported that his scalp lesions started 3 years prior to this admission and remained the same size since then. The lesions over his lower extremities started within 3 months of this admission and progressed so rapidly that it prompted him to go to the emergency department and seek help. He reported that his joint pain started approximately the same time as his scalp lesions and has progressively gotten worse over the past 3 years. He denied diarrhea, back pain, uveitis, iritis, sexually transmitted infections, abdominal pain, fever, or night sweats.

**Figure 1:**
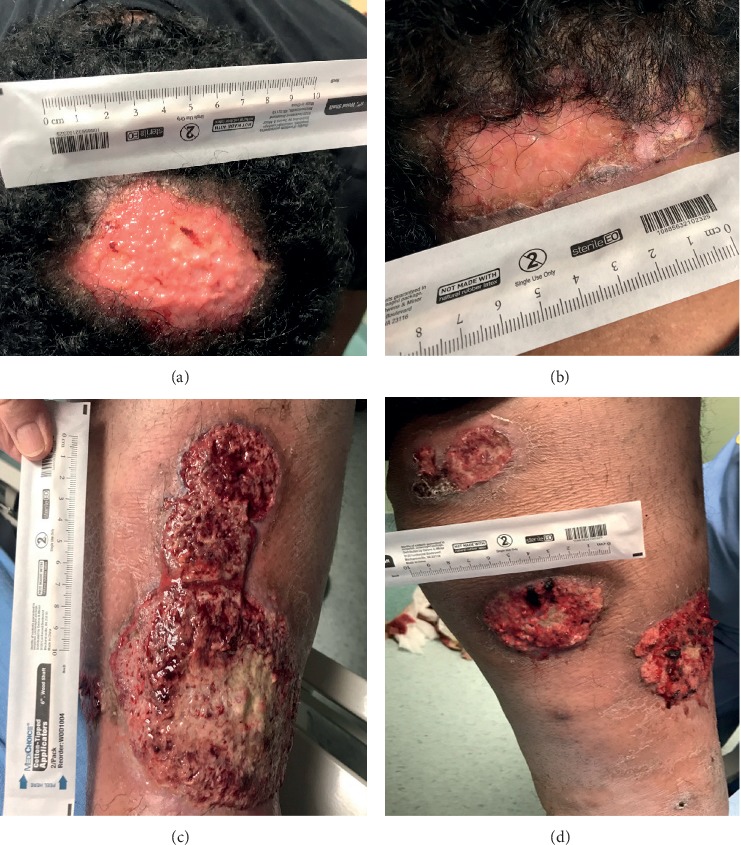
Ulcerations on initial presentation. (a) Vertex of the scalp. (b) Base of the posterior neck. (c) Right lower extremity. (d) Left lower extremity.

Physical exam was significant for a morbidly obese male in mild distress due to pain. Triage vitals were significant for mild tachycardia of 103 but otherwise were unremarkable. He had multiple wounds with serosanguineous drainage: ulcer at the vertex of the scalp measured 7 cm × 3 cm, posterior neck ulceration 5 cm × 2 cm, and multiple ulcers, of varying stages, circumferentially on bilateral lower extremities (Figure 1). Furthermore, there was tenderness and synovitis at the right 2^nd^ and 4^th^ proximal interphalangeal (PIP) joints and the left 5^th^ PIP joint. There was associated fusiform swelling of the second and fourth right digits, suggestive of dactylitis.

**Figure 2:**
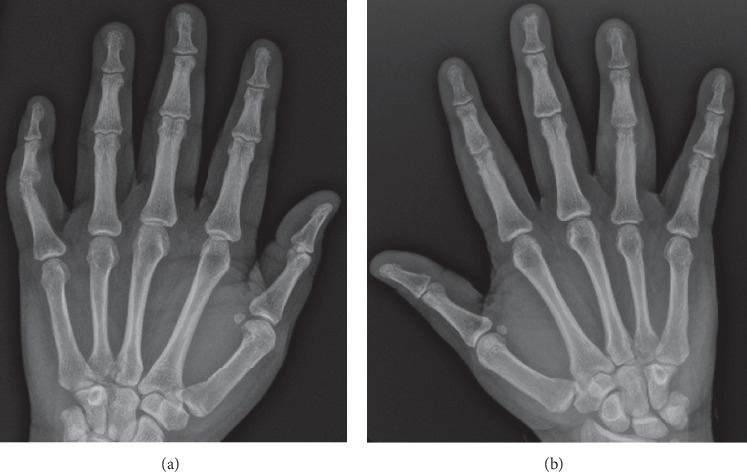
(a) X-ray of the left hand with erosive changes at the 5^th^ PIP joint. (b) X-ray of the right hand with erosive changes at the 2^nd^ and 4^th^ PIP joints.

Initial admission labs revealed leukocytosis of 12.0 × 10^3^ *μ*L (reference range 4.5–11.0 × 10^3^) with 74% neutrophils, hemoglobin of 12.4 g/dL (reference range 13.0–17.0), ESR of 43 mm/hr (reference range 0–20), and CRP 54 mg/L (reference 0.0–5 mg/L). X-ray of bilateral hands revealed marginal erosions at the right second and fourth PIP joints and left 5^th^ PIP joint (Figure 2). In view of the severity of the wounds in association with synovitis, infectious workup and rheumatological workup were initiated while he was empirically started on vancomycin and ceftriaxone.

**Figure 3:**
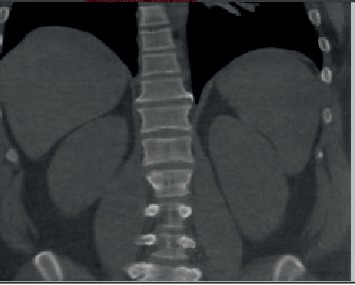
CT enterography revealing jug-like syndesmophytes at T12 and L1, suggestive of spondyloarthropathy.

Rheumatoid factor (RF), anticyclic citrullinated peptide (CCP), antinuclear antibodies (ANA), anti-Ro/SSA, anti-La/SSB, anti-RNP, complements levels, double-stranded DNA, anti-smith antibodies, anti-neutrophil cytoplasmic antibodies, HLA-B27, HIV, hepatitis B, hepatitis C, Quantiferon, and blood cultures were all negative. Punch biopsy of the left lower extremity edge revealed mixed dermal infiltrate with neutrophils and vasculitis. The tissue culture was positive for *Pseudomonas aeruginosa*. In lieu of the positive culture, he was subsequently treated for ecthyma gangrenosum with a cumulative six-week course of piperacillin/tazobactam and ciprofloxacin. Despite treatment for 6 weeks, his ulcerations did not improve. His hospital course was complicated by hematochezia, for which CT enterography and colonoscopy were completed. The colonoscopy revealed internal hemorrhoids, and small bowel biopsies were negative for findings suggestive of inflammatory bowel disease. CT enterography revealed jug-like syndesmophytes at T12 and L1 with sclerosis at the sacroiliac joints (Figure 3). The gastroenterology team planned on completing a repeat colonoscopy as an outpatient as pyoderma gangrenosum can precede inflammatory bowel disease. Ultrasound of bilateral hands revealed extensive enthesitis and erosions. Right second and fourth proximal interphalangeal (PIP) joints revealed a markedly thickened extensor tendon insertion with a mild effusion. Medial and lateral aspect of these joints revealed thickened collateral ligaments with underlying erosions and periostitis. The fifth left PIP had a similarly thickened extensor tendon insertion with periostitis and erosions medially and laterally.

**Figure 4:**
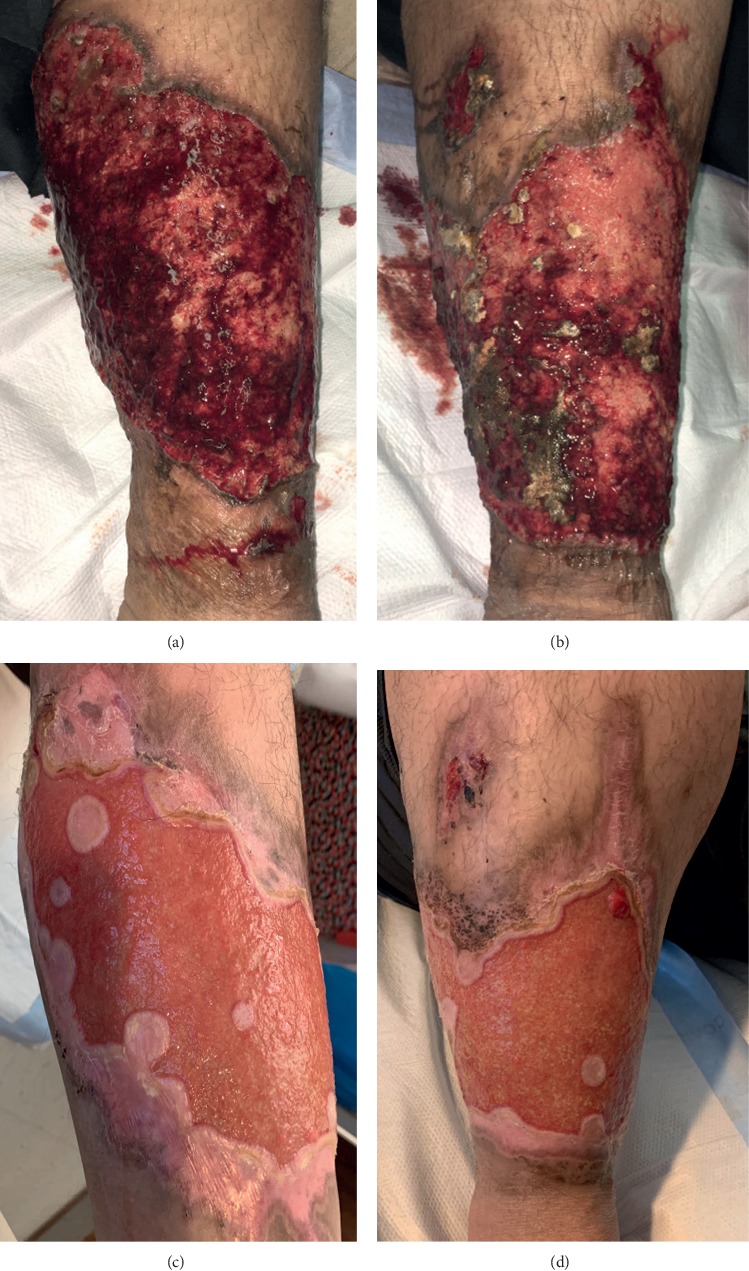
(a, b) Bilateral lower extremities prior to adalimumab initiation. (c, d) Bilateral lower extremities 4 weeks after Adalimumab treatment.

As there was no improvement of the ulcerative lesions with antibiotics, and in conjunction with syndesmophytes, enthesitis, and dactylitis, we started treatment for undifferentiated spondyloarthropathy associated with pyoderma gangrenosum. The patient was initially started on solumedrol 120 mg intravenously for 3 days followed by tapering doses of prednisone in combination with sulfasalazine. There was poor response after 6 weeks; thus, prednisone was discontinued and azathioprine was added to sulfasalazine. Azathioprine 100 mg daily and sulfasalazine 1000 mg twice daily were continued for 8 weeks with minimal response. In fact, the lower extremities ulcerations coalesced and increased in circumference. Due to lack of response to corticosteroids, sulfasalazine and azathioprine, he was subsequently started on adalimumab 40 mg subcutaneously every 14 days. Within four weeks, there was significant improvement (Figure 4). Unfortunately, we do not have pictures of complete healing as the patient moved out of state and subsequently did not follow-up. Written consent was obtained from the patient to publish his case and case-related imaging.

## 3. Discussion

Pyoderma gangrenosum can have many different clinical presentations with varying degrees of severity. Clinically, it can present as classic or ulcerative subtype, bullous, pustular, vegetative, drug-induced, postsurgical, or peristomal types [2]. Our patient had clinical manifestations compatible with classic or ulcerative pyoderma gangrenosum with two distinct phases. The ulcerative phase in this patient consisted of an initial pustule that rapidly progressed to a necrotic center with erythematous, irregular, and undermined edges, associated with severe pain. The healing stage consisted of projections of epithelium extending into the center of the ulcer, termed Gulliver's sign [2]. Gulliver's sign can be seen in Figures 4(c) and 4(d). While lower extremity involvement in pyoderma gangrenosum is common, head and neck involvement is quite rare [3]. Our patient had both lower extremity, head, and neck involvement. In fact, Ashchyan et al. completed a retrospective cohort study and found that lower extremity involvement can be found in 62% of patients, as opposed to head or neck involvement which occurs in 5% of pyoderma gangrenosum cases [3]. Despite this fact, multiple case reports have been published where head and neck involvement was the only presenting clinical feature of pyoderma gangrenosum [4–10]. In fact, Yco et al. reported a case similar to our patient with neck, lower extremity, and oral pyoderma gangrenosum in a patient with a background history of inflammatory bowel disease [10].

The exact pathophysiology of pyoderma gangrenosum is poorly understood but is believed to be a combination of genetics, irregular activation of inflammasomes, and both innate and adaptive immune systems [11]. There is a variety of disorders associated with pyoderma gangrenosum, and review of literature revealed that the most commonly associated condition is inflammatory bowel disease [2, 3, 10]. Other associated conditions with pyoderma gangrenosum include rheumatoid arthritis, seronegative arthritis, psoriatic arthritis, ankylosing spondylitis, solid organ malignancies, myelodysplasia, monoclonal gammopathy, polycythemia vera, hidradenitis suppurativa, PAPA syndrome (pyogenic arthritis, pyoderma gangrenosum, and acne syndrome), PASH syndrome (pyogenic sterile arthritis, pyoderma gangrenosum, and acne syndrome), and PA-PASH syndrome (pyogenic arthritis, pyoderma gangrenosum, acne and suppurative hidradenitis syndrome) [2]. Other case reports with suggested associations with pyoderma gangrenosum include sarcoidosis [12, 13], Sjogren's syndrome [14], systemic lupus erythematosus [15–18], Behcet's disease [19], thyroid disease [15, 20], autoimmune hepatitis [7, 21], takayasu arteritis [21], HIV [22], and cryoglobulinemia [14, 23]. Ashchyan et al. completed a cohort study of patients with pyoderma gangrenosum to determine frequency of associated conditions [3]. Specific to spondyloarthropathies, inflammatory bowel disease was associated with pyoderma gangrenosum in 41% of patients, ankylosing spondylitis in 0.6%, and psoriatic arthritis in 2.2% of patients in their cohort [3]. Unique to our case is that the patient did not have any associated inflammatory bowel disease, as colonoscopy biopsies revealed normal mucosa, and instead he had features of undifferentiated spondyloarthropathy with syndesmophytes, dactylitis, and erosive inflammatory arthritis. The incidental discovery of syndesmophytes on a CT scan did not establish a diagnosis of spondyloarthropathy in our patient; however, on ultrasound examination, there was evidence of enthesitis and periostitis at regions where our patient had severe pain and marginal erosions on X-ray. Evidence of enthesitis is a characteristic feature of spondyloarthropathy and thus helped establish a diagnosis of undifferentiated spondyloarthropathy. Inflammatory arthritis has been associated with pyoderma gangrenosum in 20 to 37 percent of cases based on different cohort studies [3, 15]. Ashchyan et al. completed a retrospective cohort study and found that a diagnosis of arthritis preceded pyoderma gangrenosum in 20% of patients [3]. Of these cases, 41% had rheumatoid arthritis, 11% had psoriatic arthritis, 3% had ankylosing spondylitis, and 45% had an unspecified inflammatory arthritis [3].

The diagnosis of pyoderma gangrenosum is challenging, as it is ultimately a diagnosis of exclusion. Su et al. have proposed diagnostic criteria for pyoderma gangrenosum and require two major and two out of four minor criteria to establish the diagnosis [24]. Major criteria include rapid progression of painful, necrolytic, cutaneous ulcer with an irregular violaceous border and exclusion of other causes of cutaneous ulceration [24]. Minor criteria include history suggestive of pathergy or clinical findings of cribriform scarring, systemic diseases associated with pyoderma gangrenosum, compatible histopathological findings, and response to treatment [24]. Our patient met both of the two major criteria, and all four of the minor criteria were proposed as diagnostic criteria by Su et al. [24]. Histopathologic findings included in these diagnostic criteria consist of sterile dermal neutrophilia with or without mixed inflammation and with or without lymphocytic vasculitis [24]. Pyoderma gangrenosum can be associated with pathergy in one-third of patients [25]. Pathergy refers to an overwhelming skin injury with minor skin trauma [25]. Our patient denied any history of trauma or surgery prior to the development of his ulcers, and thus his clinical features were not suggestive of pathergy.

Treatment of these cutaneous ulcerations is based upon the severity and the associated medical conditions. Although a direct association between severity of an associated condition and pyoderma gangrenosum has not been established, it is important that medications ideally be used that are beneficial for both conditions. Limited disease can be managed with wound care, topical, and intralesional therapy, whereas aggressive disease requires systemic immunomodulatory agents. However, no gold standard treatment exists as there are limited randomized controlled trials [25]. Topical agents can be used for ulcerations that are less than 2 cm [2], including corticosteroids, tacrolimus, sodium cromoglycate, dapsone, nicotine, and 5-aminosalicylic acid [25]. Systemic corticosteroids are first line for severe disease as either oral prednisone (0.5–1 mg/kg/day) or pulse intravenous methylprednisone 1000 mg/day [25]. Other systemic agents that can be added with steroid-sparing benefits include cyclosporine, tacrolimus, thalidomide, colchicine, dapsone, sulfasalazine, azathioprine, methotrexate, mycophenolate, cyclophosphamide, chlorambucil, minocycline, IVIG, anti-TNFs, anti-interleukin-1, anti-interleukin-12, and anti-interleukin-23 agents [25]. Our patient was given corticosteroids and then transitioned to sulfasalazine and azathioprine but did not respond. Subsequently, adalimumab was started and the patient had a drastic response within 4 weeks (Figure 4).

Initially, sulfasalazine was started for our patient's pyoderma gangrenosum as there was suspicion for an associated inflammatory bowel disease, and colonoscopy had not been completed at this point. Subsequently after minimal response, azathioprine was added as a mild immunosuppressant because our patient was considered high risk for infection with his large, open wounds. However, he did not respond. Finally, adalimumab was started instead of other longer-acting biologics such as infliximab. We preferred adalimumab as it is dosed every two weeks and thus has a shorter half-life, in case the medication had to be discontinued in the future due to an infection. Our indication for an anti-TNF agent was our patient's refractory pyoderma gangrenosum and enthesitis seen on ultrasound examination. The final diagnosis for our patient was pyoderma gangrenosum with superimposed pseudomonas infection associated with undifferentiated spondyloarthropathy.

## 4. Conclusion

Traditionally, pyoderma gangrenosum is commonly associated with inflammatory bowel disease, but this was not the case in our patient. He initially sought out treatment for ulcerations from pyoderma gangrenosum and then was subsequently found to have erosive inflammatory arthritis and undifferentiated axial spondyloarthropathy. This is suggestive of spondyloarthritis possibly going underdiagnosed and subsequently untreated in other patients with pyoderma gangrenosum. Thus, even though axial spondyloarthropathy may be rarely associated with pyoderma gangrenosum, it should be kept as an associated differential diagnosis when faced with pyoderma gangrenosum.
